# Management training in global health education: a Health Innovation Fellowship training program to bring healthcare to low-income communities in Central America

**DOI:** 10.1080/16549716.2017.1408359

**Published:** 2018-01-11

**Authors:** Andrea M. Prado, Andy A. Pearson, Nathan S. Bertelsen

**Affiliations:** a Department of Management and Organizations, INCAE Business School, San Jose, Costa Rica; b Research Department, Central American Healthcare Initiative, San Jose, Costa Rica; c Departments of Medicine and Population Health, School of Medicine, New York University, New York, USA

**Keywords:** Central America, healthcare, innovation, health management, health professionals, fellowship, training program

## Abstract

**Background**: Interprofessional education is increasingly recognized as essential for health education worldwide. Although effective management, innovation, and entrepreneurship are necessary to improve health systems, business schools have been underrepresented in global health education. Central America needs more health professionals trained in health management and innovation to respond to health disparities, especially in rural communities.

**Objective: ** This paper explores the impact of the Health Innovation Fellowship (HIF), a new training program for practicing health professionals offered jointly by the Central American Healthcare Initiative and INCAE Business School, Costa Rica. Launched in 2014, HIF’s goal is to create a network of highly trained interdisciplinary health professionals in competencies to improve health of Central American communities through better health management.

**Methods**: The program’s fellows carried out innovative healthcare projects in their local regions. The first three annual cohorts (total of 43 fellows) represented all health-related professions and sectors (private, public, and civil society) from six Central American countries. All fellows attended four 1-week, on-site modular training sessions, received ongoing mentorship, and stayed connected through formal and informal networks and webinars through which they exchange knowledge and support each other. CAHI stakeholders supported HIF financially.

**Results**: Impact evaluation of the three-year pilot training program is positive: fellows improved their health management skills and more than 50% of the projects found either financial or political support for their implementation.

**Conclusions**: HIF’s strengths include that both program leaders and trainees come from the Global South, and that HIF offers a platform to collaborate with partners in the Global North. By focusing on promoting innovation and management at a top business school in the region, HIF constitutes a novel capacity-building effort within global health education. HIF is a capacity-building effort that can be scaled up in the region and other low- and middle-income countries.

## Background

Education for health professionals in the new century is changing. According to Frenk et al. [1] three generations of educational reforms characterize progress in medical training during the past century. The first generation, launched at the beginning of the 20th century, standardized a science-based curriculum. Around midcentury, the second generation introduced problem-based instructional innovations. A third generation is now needed. These authors argue that this third generation should be competency-based, and should improve the performance of health systems by defining the core competencies that health professionals need to achieve and the ways to assess them. These competencies must be defined for specific contexts in an interdisciplinary way, and should include management skills to improve health and healthcare.

Scholars propose that this new approach be guided by two specific principles: transformative learning and interdependence in education [1]. The former would be achieved through the development of leadership attributes, while the latter would depend on opportunities for mutual learning and shared progress. The reform must emphasize the need of ‘in-patient and in-population centeredness, competency-based curriculum, interprofessional and team-based education, IT-empowered learning, and policy and management leadership skills’ [1]. Today’s health professionals must also understand the global burden of disease; recognize and improve health disparities among increasingly diverse and mobile patients and populations; and achieve competency in cross-cultural communication [2–5], The above areas provide a strong basis for designing healthcare training programs into the 21st century.

There is a shortage of health professionals worldwide [1,6]. The need to include global health disparities in medical curricula is even more relevant for training programs in developing countries, where health professionals operate in a context plagued with institutional voids and scarcity of resources. Around the world, healthcare systems are struggling with rising costs and uneven quality; thus, healthcare professionals need to maximize value for patients by pursuing the best outcomes at the lowest cost [7]. Countries in the Global South, in reference to the Brandt Line imaginary divide of all the countries in the world into the rich North and the poor South [8] face such challenges on a daily basis.

Central American countries exhibit high variation in terms of their economic and social development. Social security coverage among those formally employed also varies widely in this geographic area, with 80% coverage in Costa Rica and approximately 30% in Guatemala, Nicaragua, El Salvador and Honduras [9]. Chronic malnutrition rates among children under five capture such health disparities. For instance, Costa Rica exhibits a rate of 5.6%, while Guatemala reaches a 48%, with a region’s average of 28.4%, more than double the average in Latin America and the Caribbean [9]. An approximate 47% of the Central Americans are poor and 18% live in extreme poverty [9]. The average government investment in public health in the region is $194 per inhabitant, ranging from $714 in Costa Rica to $79 in Nicaragua [9]. Public spending on health as a percentage of GDP is on average 4.6% but it ranges from 7.6% in Costa Rica to 2.4% in Guatemala [10]. The resources currently invested in health in Central America are not sufficient to effectively serve the population’s needs and to achieve the Sustainable Development goals related to healthcare, social protection, and wellbeing proposed by United Nations. In response, the Central American Healthcare Initiative (CAHI) seeks to help overcome these health disparities and challenges in the region.

Management is essential in public health and multinational companies are increasingly represented in this sector [11]. At the community level, health systems rely on improving the organizational capacity of local organizations and strengthening interactions between institutions [12]. At the national level, multinational partnerships improve community health by inspiring leaders, sharing knowledge, engaging stakeholders, and strengthening monitoring and evaluation of programs [13]. In the private sector, business is directly leading to better health outcomes, as international development focuses more on primary healthcare, which is particularly dependent on effective health system management, and in turn improves the management of noncommunicable diseases (NCDs) that rely on well managed primary healthcare [14]. Through corporate social responsibility, corporations further impact care for chronic diseases, such as NCDs, by supporting access to medicines [15]. Although, it is recognized that management is needed for improved health and health systems, business schools are underrepresented in interprofessional health training programs [16].

Business schools bring a focus of leadership and operational skills that remain highly relevant to today’s needs in global health education [17]. By including these schools in interdisciplinary global health education, priorities in organizational design and operational competency are reinforced [18]. In addition, they are more likely to promote innovation and entrepreneurship, when compared with health education schools [19]. While competency-based health education can now be found across the spectrum of health education schools, there is no reason to exclude schools that focus primarily on effective management.

Healthcare leaders perceive change and innovation as skills necessary for professionals in the field to develop, so that organizations in this sector can face the future challenges [18]. Academics in the field of healthcare administration believe that schools are not educating students to create new processes, systems, and organizational forms, to solve problems and implement solutions across the sector. An analysis of healthcare-related curricula at 26 top US schools offering graduate degrees in healthcare administration found that the most frequently used words were *policy* and *organization* [18]. Nevertheless, academics and professionals believe it is necessary for these programs to strengthen the students’ skills on change management and entrepreneurship, two areas in which business schools can provide significant expertise. According to healthcare leaders, the goal is to create healthcare administration education more focused on innovation [18].

Global health increasingly recognizes that developed countries can learn from developing countries. Because perceptions of health vary across populations and regions, community-based approaches must define their purpose and value through locally focused perspectives of health and health needs that originate from the community itself [19]. Traditionally, vertical interventions for community health have been guided by one-size-fits-all, paternalistic approaches to implement centralized standards across diverse communities, without taking into account unique differences between communities. This approach is being replaced by horizontal solutions that originate within or are directly centered on a specific community. Given that health disparities are greater in underdeveloped communities and in developing countries, this gap provides an opportunity for health solutions that originate in developing countries to be more effective.

Lessons or best practices that Central America could share with other areas of the world include span community engagement, local healthcare worker training, financing, targeted health information, and innovative communication practices [20]. Countries in Central America can also improve their education and impact on health by creating interdisciplinary, transnational educational programs that train more-globalized health professionals to care for increasingly mobile populations of migrants and travelers [21].

## Central American Healthcare Initiative

CAHI is a coalition of Central American and US academic institutions that aims to catalyze major improvements in the quality, efficiency, and delivery of healthcare services in the Central American region by promoting innovation and better management. Robert and Elizabeth Jeffe, a philanthropic American couple living in New York City, who had previously developed a medical school in Tanzania, were interested in supporting healthcare efforts in Central America through their family foundation. Central America was geographically closer to the US than Africa and exhibited many of the greatest health challenges representative of the Global South. Faculty members at New York University and Stanford University with connections and knowledge of the region led the Jeffe Foundation to INCAE Business School, an institution founded by Harvard Business School in 1964. INCAE has two campuses, one in Costa Rica and another one in Nicaragua, but its area of influence included all the Central American countries. INCAE’s mission is ‘to actively promote the comprehensive development of the countries served, educating leaders in key sectors by improving their practices, attitudes, and values.’ This business school had strong relationships and convening power among multiple stakeholders in the region besides the private sector. INCAE often worked with local governments and civil society organizations developing strategic plans and strengthening the management skills of their members.

In 2012, CAHI and INCAE organized a local Health Summit, inviting former health ministers, social security authorities, academics, and practitioners. Organizers consulted this group of local senior leaders to determine how its intervention model could best strengthen and develop the healthcare sector in the region, and the necessary capabilities that local health professionals needed to improve healthcare access and delivery in Central America. Three proposals came out of the dialogue process, which later became CAHI’s main components: (1) a Health Innovation Fellowship (HIF), (2) a Healthcare Management Program for public hospitals’ managers, and (3) a Knowledge Center to conduct research and share best practices for effectively delivering healthcare to the poor. This paper focuses on describing and assessing the first three years of the HIF program.

CAHI’s HIF program seeks to provide the tools and support needed for social entrepreneurs to implement, scale, and replicate innovative projects that improve healthcare access for the Central Americans in the greatest need. The HIF is geared toward committed local health leaders who propose a concrete, innovative project, or idea. The chosen fellows receive training in leadership, project management, political analysis, negotiation skills, partnership management, determinants of health, and trends in global health, among other topics. They also receive mentorship from senior healthcare leaders and experts in particular fields relevant for their projects. The mentorship component pursues the strengthening of ties between senior healthcare professionals with new generations of leaders, as well as the transfer of knowledge that increases the likelihood of the projects’ success. Finally, they receive one-on-one management advice along the program, to support the development and implementation of their project in the field. In this way, the goal of HIF is to create a network of highly trained interdisciplinary health professionals in competencies to improve health of Central American communities through innovation and better management.

## Methods

CAHI selects its HIF participants based on an outline of their proposed project, its degree of innovation and its relevance to their country’s health needs. The first three cohorts (a total of 43 fellows) included representatives from multiple health-related professions, from all sectors (private, public and civil society), and from six Central American countries. The targeted populations (i.e. beneficiaries) of these projects included rural, urban, and national. Recruitment began with contacts in the public health sector and expanded through CAHI alumni networks. Having a diverse group of students increased the quality of the discussion both inside and outside the classroom, through the exchange of their country-related and professional backgrounds. CAHI fellowship projects can be categorized into four types: (1) strategies for prevention and health promotion; (2) access to health services and medications; (3) hospital management; and (4) training of health professionals (Table 1).

**Table 1.** CAHI fellows’ demographics and project types.

		Fellowship year				
		2014	2015	2016	Total	
		Fellows	Fellows	Fellows	Fellows	%
Total		16	13	14	43	100
Country						
	Guatemala	1	2	3	6	14.0
	Honduras	1	2	2	5	11.6
	El Salvador	2	1	1	4	9.3
	Nicaragua	5	1	2	8	18.6
	Costa Rica	6	5	5	16	37.2
	Panamá	1	2	1	4	9.3
Gender						
	Male	11	5	6	22	51.2
	Female	5	8	8	21	48.8
Age group						
	21–30 years	6	1	3	10	23.3
	31–40 years	4	7	4	15	34.9
	41–50 years	6	3	5	14	32.6
	51–60 years	0	1	2	3	7.0
	More than 60 years	0	1	0	1	2.3
Education background						
	Undergraduate	3	5	2	10	23.3
	Postgraduate	13	8	12	33	76.7

	Fellowship year					
	2014	2015	2016	Total		
Project type	Fellows	Fellows	Fellows	Fellows	%	Example
Strategies for prevention and health promotion	3	6	4	13	30.2	Text messages for the prevention of hypertension
						Intersectional plan for the prevention of violence, unwanted pregnancies, and chronic diseases in adolescent population
Access to medical services and prescription	5	3	2	10	23.3	Delivery of high-quality low-cost generic drugs in rural areas
						Mobile platform technology to provide remote consultations and medical care
Reinforcement of hospital management	4	2	6	12	27.9	Plan to improve processes to reduce surgery waiting list in a public hospital
						Information systems medical imaging to improve response time to diagnose patients
Training for health professionals	4	2	2	8	18.6	Specialization program focused on patient safety for nurses
						Guidelines for training health professionals in the care of indigenous populations

The HIF modular program lasts 11 months, combining on-site and online components. During the two-and-a-half-month period between on-site modules, the fellows return to their organizations but stay connected through formal and informal channels (e.g. WhatsApp group, GoToMeeting). Webinars are scheduled to complement their knowledge on specific areas. The fellows also receive a set of assignments to perform during these months, with clear deliverables and deadlines. The goal of these assignments is for fellows to implement the tools they acquire during the modules in their own projects and report on the progress made during this time.

The program uses participant-centered learning and case methodology to deliver most of its content, to develop critical thinking, and to ensure that students learn not only from their professors, but also from their classmates’ backgrounds and perspectives. The diversity of students’ backgrounds, combined with this methodology, results in a stimulating exchange of experiences between fellows, faculty, and participating organizations within the CAHI network.

The program is structured in four on-site modules, each with specific learning objectives (Table 2). Each module starts with a check-in session at which fellows present their projects and progress to their classmates, not only to receive feedback, but also to practice how to communicate effectively the main aspects of the project. The check-in session also serves as an accountability mechanism with the group and strengthens ties by providing opportunities for fellows to comment on each other’s projects. The first three modules deliver most of the program’s academic content. The fourth module wraps up the program, with the fellows presenting their projects to a panel of external jurors. Jurors consider as part of the evaluation how well the presentation delimits different dimensions of the project, such as the problem, context, necessary resources, and potential impact.

**Table 2.** HIF modular program structure.

Day 1	Day 2	Day 3	Day 4	Day 5
**Module I (Costa Rica) April 2015**				
Fellows Check-In/Presentations	Leadership For Healthcare Innovation	Leadership For Healthcare Innovation	Innovation And Healthcare	Global Health
Leadership for Healthcare Innovation	Determinants of Health	Innovation and Healthcare	Team Building	Exam/Check-Out
**Module II (Nicaragua) July 2015**				
Check-in/Strategy for Social Projects	Project Management	Leadership for Healthcare Innovation	Project Management	Global Health
Networking: It and Social Networks	Finance for Project Management	Analysis of the Impact	Project Management	Organizational Change/Exam/Check-Out
**Module III (Nicaragua) October 2015**				
Check-in/Leadership for Innovation	Effective Communication	Negotiation	Human Resource Management	Intersectorial Alliances/Replication of Social Projects
Networking: Public–Private Alliances	Political Analysis	Healthcare in Politically Unstable Countries	Health Economics	Exam/Check-Out

To achieve the above objectives, the program includes specific topics within the different modules. The first module seeks to strengthen the fellows’ leadership skills, to potentiate them as agents of change. Thus, students analyze and reflect on the role they want to play in society, as well as the challenges they would have to face to become agents of change in their countries. Materials to address this personal inquiry include historical cases of effective agents of change (e.g. Gandhi) and the analysis of significant challenges affecting humankind. The module also covers healthcare topics, such as the social determinants of health and global health needs, as well as administrative topics such as innovation management. For the latter, this module introduces students to the business model canvas [22], a pragmatic tool to help fellows ground their projects and identify the necessary building blocks to implement them effectively. These building blocks include the definition of the project’s value proposition, necessary resources, potential partners, customers (users), and costs, among others.

The second module’s main objective is to provide fellows with tools to implement different components of their projects. It includes a range of project management techniques, from dealing with unexpected events to preparing a timeline for the different steps of the process (e.g. Gantt diagram). The module also provides an overview of functional areas such as accounting, marketing, strategy, and human resource management. Finally, this module provides sessions on impact evaluation to help fellows start thinking about how they will assess the impact of their projects.

The third module encourages fellows to consider the institutional environment, which can influence the success of their project. They acquire the capabilities that they will need to perform a political and stakeholder analysis, as well as mechanisms to grow or replicate their project either directly or through partnerships. Training in negotiation and effective communication skills is also included in this module. Finally, classes teach fellows how to leverage their social networks and consider technological trends for the implementation of their projects.

In the fourth module, fellows present their projects to a panel of external jurors who evaluate their evolution. Learning assessment includes an exam at the end of each module and a final grade that evaluates participation in class and in webinars, as well as assignment and progress report delivery.

Faculty from INCAE, Stanford University, New York University, and Central American public health schools deliver the classes. Throughout the program, mentors were assigned to the fellows, often senior local healthcare professional or academics. Mentorship aims to strengthen the network of CAHI fellows and connect them with senior healthcare leaders who can share with them their experience in implementing their own projects in these countries.

To evaluate each module, and as part of INCAE’s protocol, fellows filled an electronic survey upon completion of each topic covered. At the end of the module, participants also complete a questionnaire to provide an overall assessment of the week. Additionally, to assess the HIF program, we conducted an exit survey to the fellows. The survey provides qualitative and quantitative information on the participants’ perceived benefits obtained through the program. The authors coded and analyzed the data with the SPSS statistical program to observe tendencies and generate a descriptive statistical analysis. Finally, CAHI and external researchers compared the fellows’ initial project proposals for the program with the final project presentations to the panel of jurors. This comparison allowed for an objective evaluation of the progress achieved during the 11 months.

Evaluations collected did not identify the participant, nor collected any personal health information. CAHI notified the fellows in writing that their responses would be an input to evaluate the program; that they provided consent by proceeding, and that all participation was voluntary and anonymous. This design adheres to the Institutional Review Board Ethics Committee guidelines at INCAE Business School.

## Results

**Figure 1.**
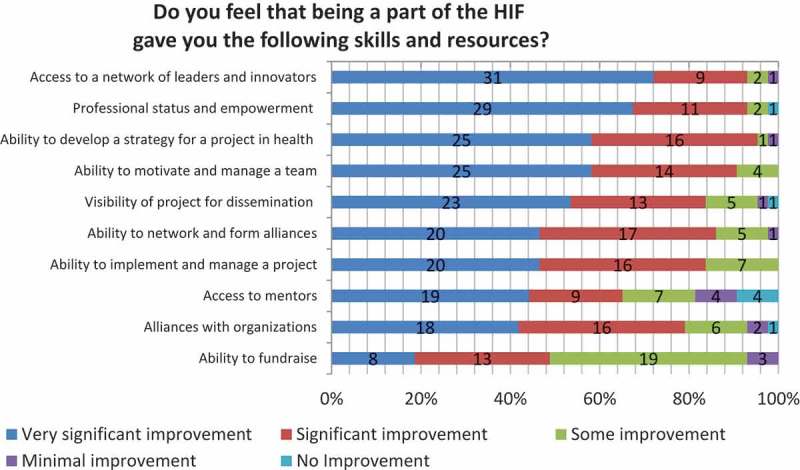
HIF impact on fellows’ skills and resources.

The assessment of the three-year pilot training program was positive at multiple dimensions. Evaluation data showed that among the perceived benefits of the program, participants noticed an improvement in their ability to develop a strategy for an innovative healthcare project and to motivate and manage team members (95% and 91%, respectively, reported significant to very significant improvement, *N* = 43). They also perceived that the program gave them access to a network of leaders and innovators, as well as professional status and empowerment (93%, respectively, reported significant to very significant improvement, *N* = 43) (Figure 1).

By comparing the application statements and the final presentations, researchers were able to analyze the evolution of the projects during the 11 months of the HIF. Applications were classified into four categories, depending on the starting point of the proposed initiative. The final reports to the jurors were also classified according to four stages. The authors coded the qualitative data, and four categories were identified: (a) idea; (b) complete business plan; (c) small-scale or pilot project; and (d) institutionalized and pursuing expansion. Institutionalized means that the organization had already embraced the project and was seeking to implement it on a larger scale.

**Figure 2.**
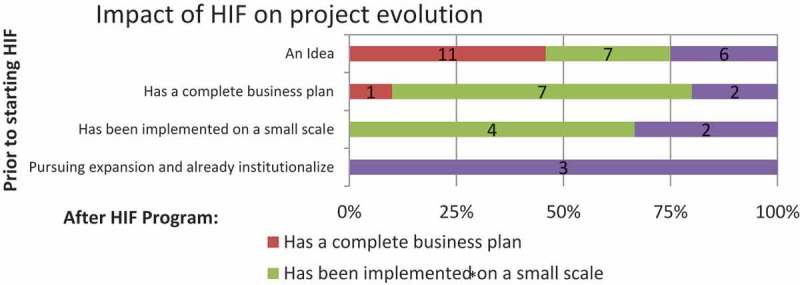
HIF impact on project evolution.

HIF contributed significantly to the development and implementation of most of the proposed initiatives. The analysis shows that out of 24 projects that started as an idea, 11 evolved into a business plan; seven were implemented on a small scale; and six became institutionalized (Figure 2). For instance, a proposal by a staff member of the Neurological Institute of Guatemala to prevent risk factors in children with intellectual disabilities started just as an idea. By graduation, the program had already trained 914 people, including children, teachers, and school directors, with the goal to train 3000 people in total by the following year. Another fellow who applied with nothing more than an idea sought to improve the distribution of supplies to a network of pharmacies that he managed in rural Nicaragua. The pharmacist developed a sophisticated business plan—including a financial and regulatory analysis—for importing, distributing, and storing low-priced and high-quality generics in the country. The above examples illustrate projects that began as ideas successfully evolved to another stage during the HIF. By the end of the HIF, both fellows had a complete business plan or were pursuing their project’s expansion.

The HIF cohorts also included fellows who applied to the program having already implemented their project on a small scale. For instance, the executive director of Teensmart, an organization that provides health education to teenagers at social risk, sought to consolidate and expand its pilot project into the Brunca region—an area with a high percentage of the country’s indigenous population and one of the poorest regions in Costa Rica. At the application stage, she was working with 10 local organizations and reaching approximately 500 teenagers. By graduation, she reported that 914 teenagers in the area were using the organization’s online platform and that Teensmart had coordinated efforts with eight additional local organizations. During HIF, she also established a strategic approach for reaching out to local organizations and for best working with them, including a fundraising strategy. The goal was to continue expanding the Teensmart operation in Costa Rica and Nicaragua, as well to apply the lessons learned in this pilot to the whole organization.

A selection of fellows’ quotes about HIF’s perceived value added in the evolution of their proposed initiatives complements this analysis. Fellows expressed these sentiments in the program’s exit survey, which was consistently positive:

- We were writing a proposal to apply for external funds. With the HIF program, we improved the focus of the objectives. Now we have a donor, partners, training, and approval of the Minister’s Office. We are ready to start.
- We were looking for collaborators outside the institution and learning about the activities of the population. Now there are three groups of instructors trained about strategies related to preventing pregnancy, suicide, and overweight.
- We had the idea of the project, but with this program, we have implemented it. Objectives are now more concrete and more realistic. We are in the implementation stage.
- We focus on decreasing turnover of healthcare professionals working in indigenous areas. At the beginning, we thought to launch the project countrywide. Now, the approach is to first conduct a pilot project in a remote geographic area using mobile technology.
- He developed the mobile application as a preliminary version (pilot) but I was not sure about its business model. Currently, I have a business plan; I revised and improved the app and it is now ready to launch in app stores. I am completing the registration process and institutionalization for the subsequent signing of strategic alliances.

**Figure 3.**
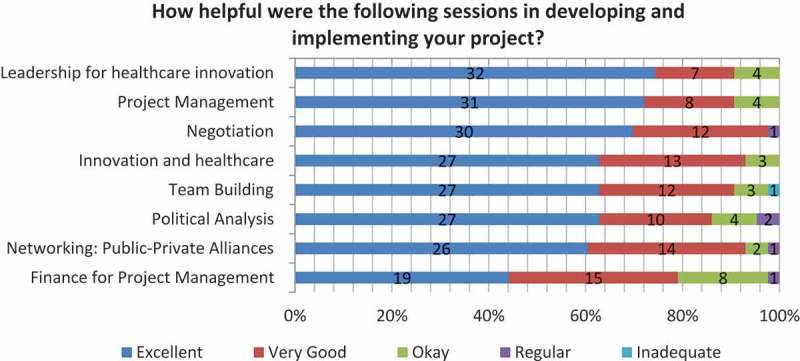
HIF impact on project development and implementation.

Reviewing specific curricular themes, the topics that received the highest positive feedback from participants were networking, public-private alliances, negotiation, innovation and healthcare (98%, 93%, and 3% respectively, reported very good or excellent, *N* = 43) (Figure 3). These results illustrate the extent to which the fellows valued the knowledge and skills that they gained—knowledge and skills that they used to increase the likelihood successfully implementing their projects.

## Discussion

The impact evaluation of the three-year pilot training program is positive: fellows improved their health management skills, and more than 50% of the projects have found either financial or political support for their implementation. By focusing on promoting innovation and management with the support of a top business school in the region, HIF constitutes a novel capacity-building effort within the domain of global health education in Central America (i.e. a region considered part of the Global South).

One strength of HIF is that most of its faculty and all its fellows come from the Global South. Additionally, HIF offers fellows a platform for collaborating with partners in the Global North—such as faculty from NYU and Stanford—leading to unique knowledge exchange. CAHI documents lessons learned by fellows in the field and shares their best practices with multiple audiences.

Fellows perceived the fact that HIF makes them part of a local network of change agents in health, as one of the most valuable contributions of the program. In this network, collaborations develop among people from different countries, who face similar challenges but address them from multiple angles and with various methods. These collaborations include mentorship (e.g. fellows sharing their expertise in a specific area); support to develop a strategy or action in a new project (e.g. replicating the project in another country or jointly developing interventions with other fellows); or participation as speakers to share experiences with new CAHI fellows. For instance, a Costa Rican fellow from the first cohort, who developed a project with mobile technologies, mentored a fellow with a project about the implementation of electronic records in a public hospital. Additionally, this fellow shared his experience implementing his project with HIF’s third cohort participants, in an event organized by CAHI. Such networking practices aim at strengthening knowledge exchange and development of ties among fellows. This example shows that HIF constitutes not only a North–South knowledge exchange platform, but also a South–South platform.

Despite its initial positive results, HIF also has opportunities for improvement. For example, an 11-month period program is a relatively short time to generate significant improvements in the health system and to evaluate the outcomes of each initiative. Measuring the impact of many of these projects requires specialized techniques (e.g. randomized control trials) and a longer-term horizon. In this regard, the multiple health issues addressed by the fellows (e.g. nutrition; waiting lists in hospitals; women’s reproductive health; training of community health promoters) makes it difficult to standardize the impact evaluation process and the development of appropriate indicators. Moreover, the fact that projects are not all at the same development stage when submitted for the HIF application process makes it difficult to use parameters to compare them.

## Conclusion

This preliminary impact evaluation of HIF is positive and encouraging. Nevertheless, Central America and the Global South in general have multiple challenges to face. Change must happen faster if we are to achieve the Sustainable Development Goals by 2030. HIF might explore increasing the number of participants per cohort without jeopardizing the quality of the program, particularly the follow-up process of the fellows’ projects.

We believe it is necessary for programs like HIF and initiatives working with local leaders to question whether they are providing the necessary conditions for health workers with limited formal education (e.g. without a university degree) to benefit from such training. Having community health promoters in the cohort would not only bring diversity to the group but also enrich the discussion. It is valuable for hospital directors and physicians to understand the reality that these community leaders face every day on the ground. Although HIF included some local leaders in the program, the strengths of having community health workers in each cohort cannot be emphasized enough. While basic health services in rural areas certainly needed more physicians, nurses, and pharmacists, fellows that worked with community health workers are likely to reach the poorest populations in a shorter term. This point is acknowledged in the field of task shifting, as bringing healthcare to the poorest in the region requires the development and engagement of community leaders to deliver basic healthcare services. Thus, CAHI might explore the possibility of launching a *Train the Trainers* program, focused on training an army of these leaders who could have a faster and more direct impact on the populations that need it the most. In this way, strengthening the promotion of innovation and management training among community health workers could help reduce global health disparities and achieve the Sustainable Development Goals in Central America.

## Paper context

Global health education is increasingly interdisciplinary, and global health interventions focus heavily on health system improvements. Here, we believe this approach to train health professionals in health management skills with a fellowship hosted at a local business school—in partnership with two US medical schools—is an innovative and impactful program, which in turn strengthens local responses to their communities’ health needs in rural Central America.
